# Intranasal delivery of VEGF enhances compensatory lung growth in mice

**DOI:** 10.1371/journal.pone.0198700

**Published:** 2018-06-07

**Authors:** Duy T. Dao, Jacqueline T. Vuong, Lorenzo Anez-Bustillos, Amy Pan, Paul D. Mitchell, Gillian L. Fell, Meredith A. Baker, Diane R. Bielenberg, Mark Puder

**Affiliations:** 1 Vascular Biology Program, Boston Children’s Hospital, Boston, Massachusetts, United States of America; 2 Department of Surgery, Boston Children’s Hospital, Boston, Massachusetts, United States of America; 3 Institutional Centers for Clinical and Translational Research, Boston Children’s Hospital, Boston, Massachusetts, United States of America; National Yang-Ming University, TAIWAN

## Abstract

Vascular endothelial growth factor (VEGF) has previously been demonstrated to accelerate compensatory lung growth (CLG) in mice and may be a useful therapy for pulmonary hypoplasia. Systemic administration of VEGF can result in side effects such as hypotension and edema. The aim of this study was to explore nasal delivery as a route for intrapulmonary VEGF administration. Eight-week-old C57BL/6 male mice underwent left pneumonectomy, followed by daily nasal instillation of VEGF at 0.5 mg/kg or isovolumetric saline. Lung volume measurement, morphometric analysis, and protein expression studies were performed on lung tissues harvested on postoperative day (POD) 4. To understand the mechanism by which VEGF accelerates lung growth, proliferation of human bronchial epithelial cells (HBEC) was assessed in a co-culture model with lung microvascular endothelial cells (HMVEC-L) treated with and without VEGF (10 ng/mL). The assay was then repeated with a heparin-binding EGF-like growth factor (HB-EGF) neutralizing antibody ranging from 0.5–50 μg/mL. Compared to control mice, the VEGF-treated group displayed significantly higher lung volume (*P* = 0.001) and alveolar count (*P* = 0.005) on POD 4. VEGF treatment resulted in increased pulmonary expression of HB-EGF (*P* = 0.02). VEGF-treated HMVEC-L increased HBEC proliferation (*P* = 0.002) while the addition of an HB-EGF neutralizing antibody at 5 and 50 μg/mL abolished this effect (*P* = 0.01 and 0.002, respectively). These findings demonstrate that nasal delivery of VEGF enhanced CLG. These effects could be mediated by a paracrine mechanism through upregulation of HB-EGF, an epithelial cell mitogen.

## Introduction

Vascular endothelial growth factor (VEGF) is an endothelial cell mitogen that serves as a key regulator of angiogenesis and neovascularization [1]. It is essential for the normal development and regeneration of human tissues and organs [2–4]. The absence of VEGF signaling has devastating effects on fetal lung development and its neutralization is associated with decreased lung maturation, surfactant production, and capillary and alveolar hypoplasia in animal models [5,6]. In addition, low levels of VEGF have been observed in human and animal models of infantile diseases such as respiratory distress syndrome and pulmonary hypoplasia in congenital diaphragmatic hernia (CDH) [7,8]. Conversely, increased expression of VEGF through gene therapy increases lung angiogenesis and promote alveolar growth in hyperoxia-induced injury in rat lungs [9]. Results from these studies support the hypothesis that there may be a potential role for VEGF in mediating regenerative lung growth.

Unilateral pneumonectomy (PNX) results in compensatory growth of the remaining lung in mammalian species such as mouse, rat, and swine [10–12]. This process recapitulates the late alveolar stage of postnatal lung growth [10]. Our group has previously shown that systemic administration of VEGF accelerates compensatory lung growth (CLG) in mice, resulting in a return to baseline lung volume in 4 days with VEGF treatment as compared to 8–10 days in saline-treated mice [13]. This feature renders VEGF a promising therapy for the treatment of neonatal hypoplastic lung diseases. However, systemic administration of VEGF may result in undesirable side effects if used in humans. Past clinical studies of the potential therapeutic uses of VEGF in critical limb ischemia and coronary artery disease noted side effects such as hypotension and decreased cardiac stroke volume [14–16]. In addition, VEGF promotes vascular permeability [17] and elevated levels of VEGF have been associated with skeletal muscle and macular edema [18]. Systemic VEGF administration may also exacerbate pathogenic angiogenesis, such as that seen in diabetic retinopathy [19] and cancer metastasis [20]. Given the possible adverse systemic effects, the ability to locally administer VEGF for potential therapeutic purposes would therefore be advantageous.

Nasal delivery offers a less invasive and more specific alternative to systemic administration, reducing opportunities for the development of side effects. Nasal instillation has been studied for delivery of therapeutics to the brain and the lungs [21]. When instilled intranasally, 75% of dye deposits to the airways, with no detectable amounts in non-targeted areas, such as the stomach or esophagus [22]. In our current study, we proposed topical administration of exogenous murine VEGF through nasal instillation is a comparable alternative to systemic VEGF administration. The mechanisms by which VEGF accelerates CLG were also explored with *in vitro* studies utilizing lung endothelial and epithelial cells.

## Materials and methods

### Comparison of plasma VEGF levels between systemic and topical administration

C57BL/6 mice at 8 weeks of age were administered recombinant murine VEGF_164_ (GenScript, Piscataway, NJ) at 0.5 mg/kg via either intraperitoneal (ip) injection or nasal instillation (N = 4 in each group). For nasal instillation, mice were sedated with isoflurane until a regular respiratory rate of 50–70 breaths/min was achieved. The mouse was held upright and a droplet of concentrated VEGF_164_ (200 μg/mL) was pipetted onto its nose at a volume dose of 2.5 μL/g.

To quantify the amount of systemic absorption of VEGF with each route of administration, blood collection was performed via retro-orbital puncture 5 minutes after VEGF administration for the baseline timepoint, and repeated at 30, 120, and 240 minutes. Blood was collected in EDTA-containing collection tubes and plasma was separated by centrifugation at 2000 g and 4 °C for 15 minutes. Plasma VEGF levels were measured with an enzyme-linked immunosorbent assay (ELISA) (R&D Systems, Minneapolis, MN) according to the manufacturer’s protocol.

### Surgical model and experimental groups

Eight-week-old C57BL/6 male mice (Jackson Laboratories, Bar Harbor, ME) underwent left PNX as previously described [23]. Animals were anesthetized with 120–400 mg/kg of Avertin (Sigma, St. Louis, MO) via intraperitoneal injection and orotracheally intubated. After the left chest was prepared with ethanol and iodine solution, a skin incision was made along the left anterior axillary line. The left lung was accessed via a thoracotomy incision at the fifth intercostal space and the hilum was ligated with a 4–0 silk suture (Ethicon, Somerville, NJ). Thoracotomy and skin incisions were closed with running PDS^®^ (Ethicon, Somerville, NJ), followed by resuscitation with 3 mL of subcutaneous warm normal saline. Buprenorphine was administered post-operatively twice daily via subcutaneous injection for 72 hours. Following a left PNX, mice were randomized into either the control or VEGF group. The VEGF group received 0.5 mg/kg/day of VEGF_164_ via nasal instillation as previously described while control mice received an isovolumetric volume of normal saline. All procedures were carried out according to the National Institutes of Health Guide for the Care and Use of Laboratory Animals and approved by the Institutional Animal Care and Use Committee at Boston Children’s Hospital.

### Pulmonary function tests and physical activity assessment

On post-operative day (POD) 4, mice (N = 13 in each group) were anesthetized with Avertin as previously described, followed by a midline neck incision to gain access to the trachea. A tracheotomy was performed and the trachea was cannulated with a 20-gauge hollow bore needle, which was subsequently connected to a Flexivent^®^ system (SCIREQ, Montreal, Canada). Total lung capacity and pulmonary compliance were derived from the pressure-volume loops. All parameters were normalized for body weight. Pulmonary function was assessed on POD 4 as this was the point of most active lung growth according to our previous work [24].

On POD 10, another subset of mice (N = 5 VEGF and N = 5 control) were placed in a 42 x 42 cm open field where their traveled distance, rest time, and basic and fine movements were recorded for 6 minutes with an infrared-based motion tracking system (Motormonitor, Kinderscientific, Poway, CA). Fine movements referred to instances when the animal broke the same beam of infrared without moving from its spot, e.g. grooming or head movements, while basic movements captured the number of times the animal blocked a new beam. Mice were then placed on a rodent treadmill (IITC Life Science, Woodland Hills, CA) for compulsory exercise. The exercise regimen included 5 minutes of habituation at 3 cm/s, followed by 10 minutes of gradually increasing speed from 3 cm/s to 10 cm/s. Following exercise, mice were placed back into the open field for post-treadmill activity measurements. Physical activity was assessed on POD 10 to avoid the potentially confounding effects of post-operative pain that may still be present on POD 4.

### Lung volume measurement and morphometric analyses

Animals were euthanized with Avertin after functional studies as previously described on POD 4. The remaining right lung was removed and inflated with 10% formalin at 30 cmH_2_O. Lung volume was measured with the water displacement method [25] and normalized for body weight (N = 10 in each group). Of note, 26 mice (13 in each group) underwent functional studies, yet lung volume measurement was only performed in 20 animals (10 in each group). The remaining lung specimens were inadvertently injured during organ harvest, which precluded formalin inflation. No lung specimens were excluded for any other reason. The trachea was then ligated with a silk suture and the specimen was preserved in 10% formalin at 4°C for 24 hours before being transferred to 70% ethanol. All specimens were subsequently embedded in paraffin for histologic analyses. Lung volume was similarly measured on POD 10 (N = 7 control and N = 5 VEGF).

Hematoxylin and eosin (H&E)-stained sections from lung specimens harvested on POD 4 (N = 5 in each group) were used for morphometric analyses based on the principle of systemic uniform random sampling [26,27]. At least 40 non-overlapping lung fields were examined at 200X magnification for each section. Point and intersection counting was performed on a 42-point test lattice superimposed onto each lung field. Parameters generated by this technique included parenchymal volume, alveolar volume, septal surface area, and mean septal thickness. Parenchyma was defined as the respiratory region of the lung, which included alveoli, terminal ducts, and alveolar septa. Alveolar density was determined by counting the number of alveolar transections on randomly chosen lung fields using the method of Weibel and Gomez [28]. The total alveolar number was the product of alveolar density and parenchymal volume. All volume and area measurements were normalized for body weight.

### Protein expression analyses

#### Pulmonary levels of VEGF

Tissue levels of pulmonary VEGF on POD 4 were determined with ELISA (R&D Systems, Minneapolis, MN) according to the manufacturer’s protocol. Briefly, lung tissue was homogenized in radioimmunoprecipitation assay (RIPA) buffer (Boston Bio Products, Ashland, MA) containing protease and phosphatase inhibitors (Thermo Fisher Scientific, Waltham, MA). After centrifugation for 15 minutes at 4°C and 14,000 rpm, the supernatant was collected and total protein concentration was determined with a Bradford colorimetric assay (Bio-Rad Laboratories, Hercules, CA). The extract was then added to the supplied ELISA plate and incubated for 2 hours at room temperature. This was followed by horseradish peroxidase (HRP) conjugation for another 2 hours and addition of chemiluminescent reagent. The chemiluminescent signal was detected at 450 nm and the protein concentration was determined from a standard curve. Three biological replicates were used for each experimental group and VEGF concentration was normalized against total protein concentration.

#### Immunoblot

Proteins were extracted from lung tissues harvested on POD 4 as previously described. Forty micrograms were mixed with Laemmli buffer (Boston BioProducts, Ashland, MA) and heated to 95°C for 5 minutes. Proteins were separated on a 4–12% Bis-Tris polyacrylamide gel (Thermo Fisher Scientific, Waltham, MA). The separated proteins were subsequently transferred to a nitrocellulose membrane and blocked in 5% non-fat dry milk (Bio-Rad Laboratories Inc., Hercules, CA) for 1 hour at room temperature. The membranes were incubated for 24 hours at 4°C in primary antibodies, anti-P-VEGFR2, -VEGFR2, -P-EGFR, -EGFR (Cell Signaling Technology, Danvers, MA), -HB-EGF (R&D Systems, Minneapolis, MN), and -β-Actin (Sigma-Aldrich, St. Louis, MO). Next, the membrane was washed with Tris-buffered saline and Tween 20 (TBST) (Boston BioProducts, Ashland, MA) before being incubated for 1 hour at room temperature with HRP-conjugated goat anti-rabbit or anti-mouse secondary antibodies (Santa Cruz Biotechnology, Dallas, TX). The membrane was developed with enhanced chemiluminescence (ECL) reagents (Thermo Fisher Scientific, Waltham, MA) and the signal was exposed using ChemiDoc Touch (BioRad, Hercules, CA). Three biological replicates from the control group and four from the VEGF group were analyzed.

#### Immunohistochemistry

Immunohistochemistry (IHC) was performed on formalin-fixed, paraffin-embedded lung sections dried in an oven at 58 °C. All sections were deparaffinized with xylene and progressively rehydrated in various concentrations of ethanol. Heat-induced epitope retrieval was achieved with a citrate-based unmasking solution (Vector Laboratories, Burlingame, CA) at 120 °C in a pressurized chamber (Decloaking Chamber^™^, Biocare Medical, Pacheco, CA). Slides were washed with PBST (phosphate-buffered saline with 0.5% Triton-X) for 10 minutes x3, followed by 30 minutes of incubation in blocking solution (PBST containing 1% bovine-serum albumin). Incubation with primary antibodies was done overnight at 4 °C. Primary antibodies were prepared in blocking solution and included rat anti-Ki67 (Invitrogen, Carlsbad, CA) and rabbit anti-ERG (Abcam, Cambridge, MA) antibodies. ERG is a nuclear marker of endothelial cells and was used to facilitate cell counting [29]. On the next day, slides were again washed with PBST for 30 minutes x3, followed by incubation in secondary antibodies, Alexa Fluor-conjugated donkey anti-rat (Abcam, Cambridge, MA) and anti-rabbit (Invitrogen, Carlsbad, CA) IgG antibodies. DAPI counterstaining was performed for 5 minutes. Slides were washed again with PBST, dried, and mounted.

Sections of control and VEGF-treated lungs harvested on POD 2 (N = 5 in each group) and 4 (N = 4 control and N = 5 VEGF) were examined with a confocal microscope (LSM 800, Zeiss, Jena, Germany) at 10X and 20X magnification. For each specimen, cell counting was performed on four random fields sampled across the entire right lung at 10X magnification. Endothelial cells were quantified with ImageJ by counting ERG-positive cells. Proliferating endothelial cells, marked by double staining with ERG and Ki67, were counted manually. Percent proliferating endothelial cells was calculated by normalizing the number of proliferating endothelial cells against total endothelial cells.

### Quantitative polymerase chain reactions (qPCR)

Total RNA was extracted from lung tissues harvested on POD 4 with the RNeasy Mini Kit (Qiagen, Hilden, Germany) according to the manufacturer’s protocol. Amplification reactions were performed with the StepOne Real-Time PCR Systems (Applied Biosystems, Foster City, CA) with TaqMan^®^ primers for VEGF receptor (VEGFR) 1, VEGFR2, and VEGF (Applied Biosystems, Foster City, CA). All target genes were normalized to the housekeeping gene GAPDH and their mRNA levels calculated using the 2^ΔΔCt^ method [30]. Three biological replicates from the control group and four from the VEGF group were used for the reactions.

### Co-culture of lung endothelial and epithelial cells

#### Angiogenesis and protease array

First, human pulmonary microvascular endothelial cells (HMVEC-L) (Lonza, Allendale, NJ) were lysed on ice with Laemmli buffer after 5 minutes of treatment with human VEGF_165_ (GenScript, Piscataway, NJ) at 10 ng/mL and activation of VEGFR2 was confirmed with immunoblot for P-VEGFR2.

Once activation was confirmed, HMVEC-L were treated without (control) or with VEGF_165_ for 8 hours. Protein content of conditioned media from control and VEGF-treated HMVEC-L were analyzed with a Proteome Profiler^™^ Angiogenesis Array (R&D Systems, Minneapolis, MN) while cell lysate was characterized with a Protease Array (R&D Systems) according to the manufacturer’s protocol. Briefly, nitrocellulose membranes embedded with capture antibodies were blocked for 1 hour prior to the addition of conditioned medium or cell lysate samples. Samples were incubated with an antibody cocktail for 1 hour at room temperature before being applied on the membranes for overnight incubation at 4°C. After conjugation with streptavidin-HRP, signals were developed with a hydrogen peroxide and luminol-based reagent mix and quantified using ImageJ (NIH, Bethesda, MD).

#### Proliferation assay

HMVEC-L and human bronchial epithelial cells (HBEC) (ATCC, Manasses, VA) were cultured under aseptic technique at 37 °C. First, HBEC were grown to 50% confluence on a 24-well plate, followed by 24-hour starvation in airway epithelial cell basal medium (ATCC, Manassas, Virginia) with human serum albumin 500 μg/mL, linoleic acid 0.6 μM, lecithin 0.6 μg/mL, and L-glutamine 6 mM. Half of the wells were then treated with VEGF_165_ at 10 ng/mL while the other half received no treatment. Cell viability was assessed at baseline and every 24 hours for 72 hours with the WST-8 cell counting kit (Sigma Aldrich, St. Louis, MO). The assay was done in quadruplicates.

Subsequently, co-culture of HMVEC-L and HBEC was performed with Transwell^®^ permeable inserts (Corning, Corning, NY) containing 0.4 μm pores. HMVEC-L were plated on the inserts and expanded until confluence before being starved for 24 hours in endothelial basal medium 2 (EBM-2) (Lonza, Allendale, NJ) + 0.5% fetal bovine serum (FBS). Meanwhile, HBEC were expanded to 50% confluence and starved as previously described. On the day of treatment, half of HMVEC-L-covered inserts were treated with VEGF_165_ at 10 ng/mL. The other half was the control and received no treatment. The inserts were subsequently transferred to the 24-well plate containing HBEC. Cell viability was assessed at baseline and every 24 hours for 72 hours as previously described. The assay was done in quadruplicates.

#### HB-EGF neutralization assay

The proliferation assay was repeated with the addition of an HB-EGF neutralization antibody (R&D Systems). VEGF (10 ng/mL) and HB-EGF neutralizing antibody were separately added to chambers containing HMVEC-L and HBEC, respectively. Five conditions were tested: +VEGF/-antibody (Ab), -VEGF/+Ab 5 μg/mL, +VEGF/+Ab 0.5 μg/mL, +VEGF/+Ab 5 μg/mL, and +VEGF/+Ab 50 μg/mL. Cell viability was assessed after 24 hours of treatment as previously described. The assay was done in quadruplicates.

To confirm the neutralizing effect of HB-EGF antibody, HBEC were lysed with Laemmli buffer after 24 hours of treatment and the degree of EGFR activation was assessed with immunoblot of P-EGFR and EGFR (Cell Signaling Technology). Two treatment conditions were compared, +VEGF/-Ab and +VEGF/+Ab 5 μg/mL. The experiment was done in quadruplicates.

### Statistical analyses

The area under the curve (AUC) was calculated from the curves generated from plasma levels of VEGF at different timepoints following intranasal and ip administration. Comparison of AUC between the two delivery methods was achieved with a two-sided Student’s *t*-test. Lung volume adjusted for body weight was analyzed with a two-way analysis of variance (ANOVA) using Sidek correction for multiple comparisons. Tests of significance for pulmonary function tests, morphometric analyses, qPCR, ELISA, percent proliferating endothelial cells, and immunoblot between the control and VEGF groups were performed with two-sided Student’s *t*-test. A paired *t*-test was used to analyze pre- and post-treadmill parameters on physical activity assessments. Comparisons of cell density at various time points on proliferation assays was achieved with multiple *t*-tests and Sidak-Bonferroni correction for multiple comparisons. For HB-EGF neutralization assays, a one-way ANOVA with Dunnett correction for multiple comparisons was used to compare cell density of different conditions to +VEGF/-Ab. A *P* value of < 0.05 was considered statistically significant. Results are presented as mean ± standard error (SE). All analyses were performed with GraphPAD Prism v.7.

## Results

### Nasal instillation of VEGF improved lung volume, pulmonary function, and physical activity in mice after left PNX

**Fig 1:**
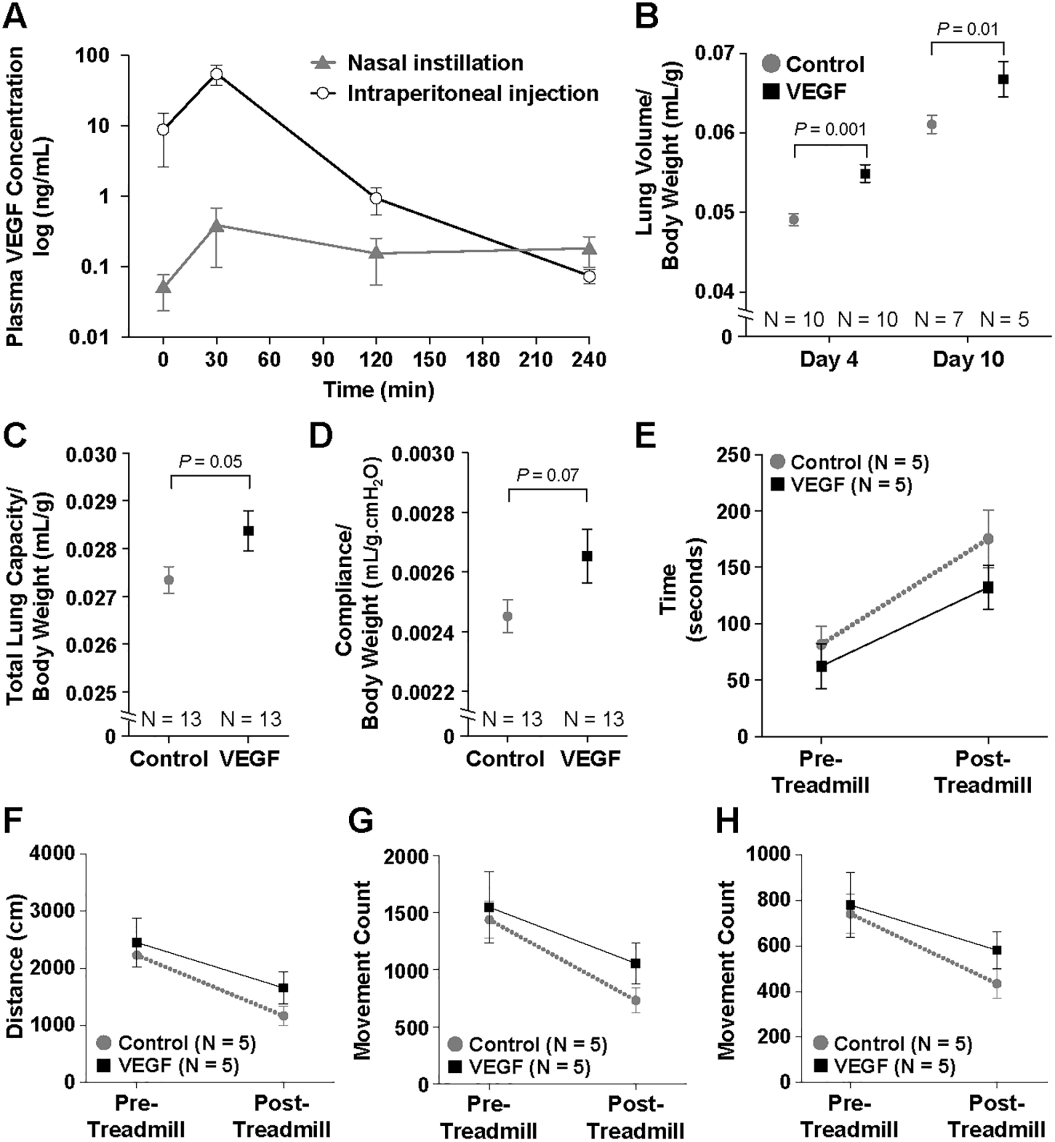
Lung volume and functional assessments. Nasal instillation resulted in less systemic absorption of VEGF compared to intraperitoneal injection as measured by enzyme-linked immunosorbent assay (ELISA) (A). VEGF-treated mice display higher post-euthanasia lung volume on post-operative day (POD) 4 and 10 (B). There is a trend toward increased total lung capacity (C) and pulmonary compliance (D) with VEGF treatment on POD 4. Although not reaching statistical significance, mice in the VEGF group performed better on post-treadmill rest time (E), walking distance (F), basic movement count (G), and fine movement count (H) than the control mice on POD 10. Data are expressed as mean ± standard error of the mean (SE).

First, to compare the degree of VEGF absorption between ip injection and nasal instillation, plasma VEGF concentration was measured at different timepoints after administration. While there was a low and steady level of VEGF detected in the plasma after nasal instillation, ip injection resulted in a spike of VEGF concentration at 30 minutes, followed by a rapid decline (Fig 1A). Overall, the total AUC with ip injection was almost 70 times higher than that of nasal instillation (3529 ± 830 vs 51 ± 16 min.ng/mL, *P* = 0.003).

To test the effect of topically delivered VEGF in a model of CLG, mice underwent left PNX and were randomized to receive either saline or VEGF via nasal instillation daily. Post-euthanasia body weight-normalized lung volume in the VEGF group was significantly higher than that of the control mice on both POD 4 (5.49 ± 0.11 vs 4.91 ± 0.07 x 10^−2^ mL/g, *P* = 0.001) and POD 10 (6.68 ± 0.22 vs 6.11 ± 0.11 x 10^−2^ mL/g, *P* = 0.01) (Fig 1B). Pulmonary function measured on POD 4 before euthanasia also showed a trend towards improvement in the VEGF group. VEGF-treated mice displayed improved total lung capacity (2.84 ± 0.04 vs 2.73 ± 0.03 x 10^−2^ mL/g, *P* = 0.05) (Fig 1C) and pulmonary compliance (2.65 ± 0.09 vs 2.45 ± 0.06 x 10^−3^ mL/g.cmH_2_O, *P* = 0.07) (Fig 1D).

On POD 10, five randomly selected mice in each group underwent physical activity measurements before and after a compulsory treadmill exercise regimen. Although not reaching statistical significance, the VEGF group performed better across all parameters in the post-treadmill period, including longer walking distance, reduced rest time, and higher basic and fine movement counts (Fig 1E–1H). Overall, topical VEGF enhanced lung growth and potentially improved pulmonary function after left pneumonectomy.

### VEGF-treated lungs displayed increased alveolarization and endothelial proliferation

**Fig 2:**
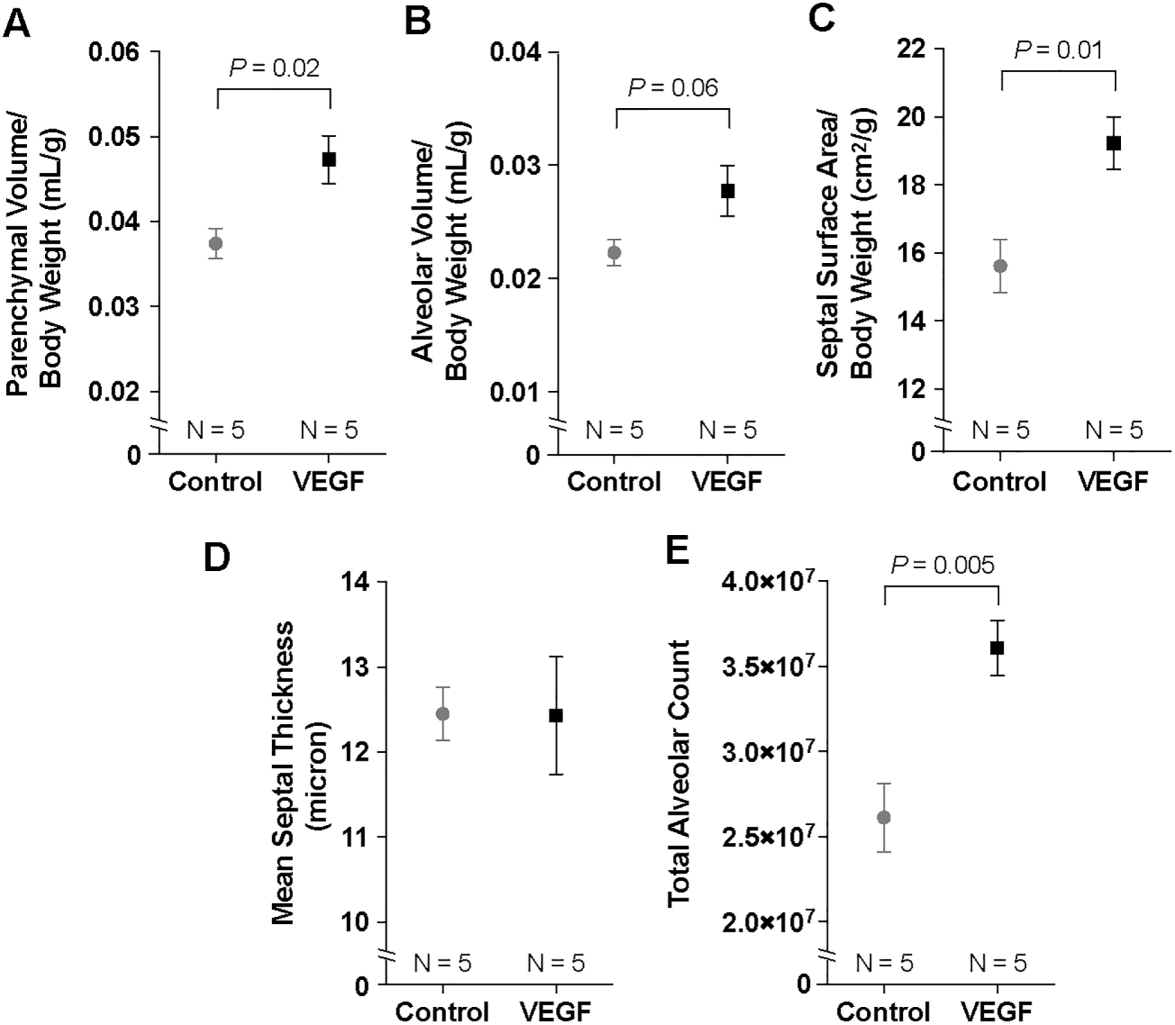
Morphometric analyses. Lungs of VEGF-treated mice demonstrate increased parenchymal volume (A) and a trend toward higher alveolar volume (B). Septal surface area (C) was higher in the VEGF group but there was no difference in mean septal thickness (D). VEGF-treated mice had increased total alveolar count (E). Data are expressed as mean ± SE.

Morphometric analyses were performed with a point and intersection counting technique. On POD 4, VEGF-treated lungs demonstrated increased normalized parenchymal volume (4.71 ± 0.28 vs 3.74 ± 0.18 x 10^−2^ mL/g, *P* = 0.02) (Fig 2A), septal surface area (19.2 ± 0.8 vs 15.6 ± 0.8 cm^2^/g, *P* = 0.01) (Fig 2C), and total alveolar count (3.61 ± 0.16 vs 2.61 ± 0.20 x 10^7^, *P* = 0.005) (Fig 2E). There was also a trend toward increased alveolar volume in the VEGF group (2.77 ± 0.22 vs 2.23 ± 0.11 x 10^−2^ mL/g, *P* = 0.06) (Fig 2B). Of note, there was no difference in mean septal thickness between the two groups (Fig 2D), indicating an absence of pulmonary edema.

**Fig 3:**
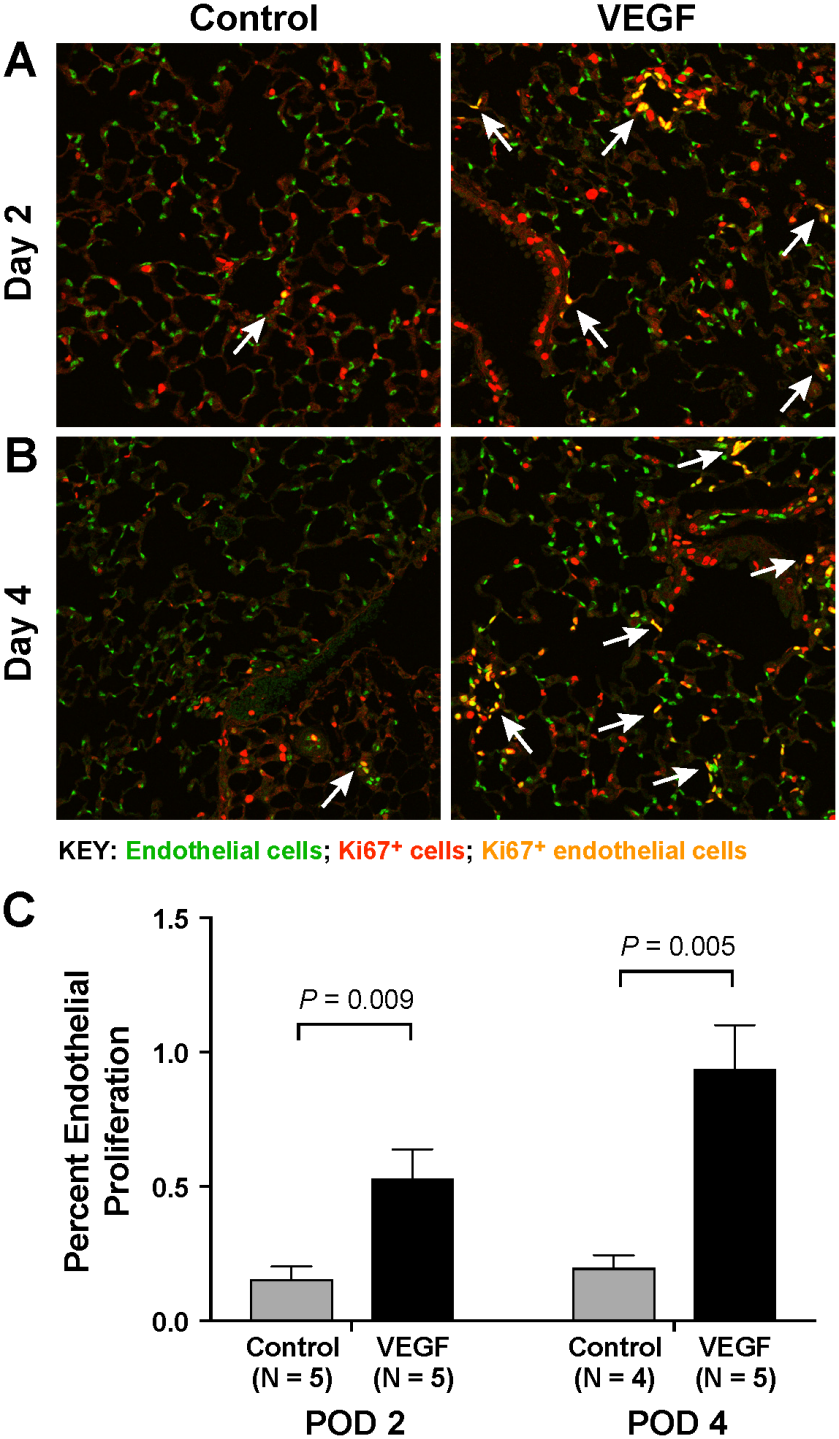
Immunohistochemistry. Lung endothelial cells are labeled with anti-ERG antibody (green) and proliferating cells are marked with anti-Ki67 antibody (red). Double-stained Ki67-positive endothelial cells appear yellow (arrows). Representative sections of control and VEGF-treated lungs harvested on POD 2 (A) and 4 (B) are shown at 20X magnification. Topical VEGF treatment significantly increases endothelial proliferation on both POD 2 and 4 (C).

In order to assess the effect of VEGF on lung endothelial cells, nuclear staining of lung endothelial cells was performed to facilitate counting (Fig 3A and 3B). Topical VEGF treatment significantly increased percent proliferating endothelial cells on both POD 2 (0.54 ± 0.10 vs 0.16 ± 0.04, *P* = 0.009) and 4 (0.94 ± 0.16 vs 0.20 ± 0.04, *P* = 0.005) (Fig 3C). Proliferating endothelial cells assumed a clustering pattern. However, these clusters were distributed equally throughout the right lung and there was no predilection for a central or peripheral region.

### Nasal instillation of VEGF delivered the agent to the lungs and increased VEGFR2 and HB-EGF levels

**Fig 4:**
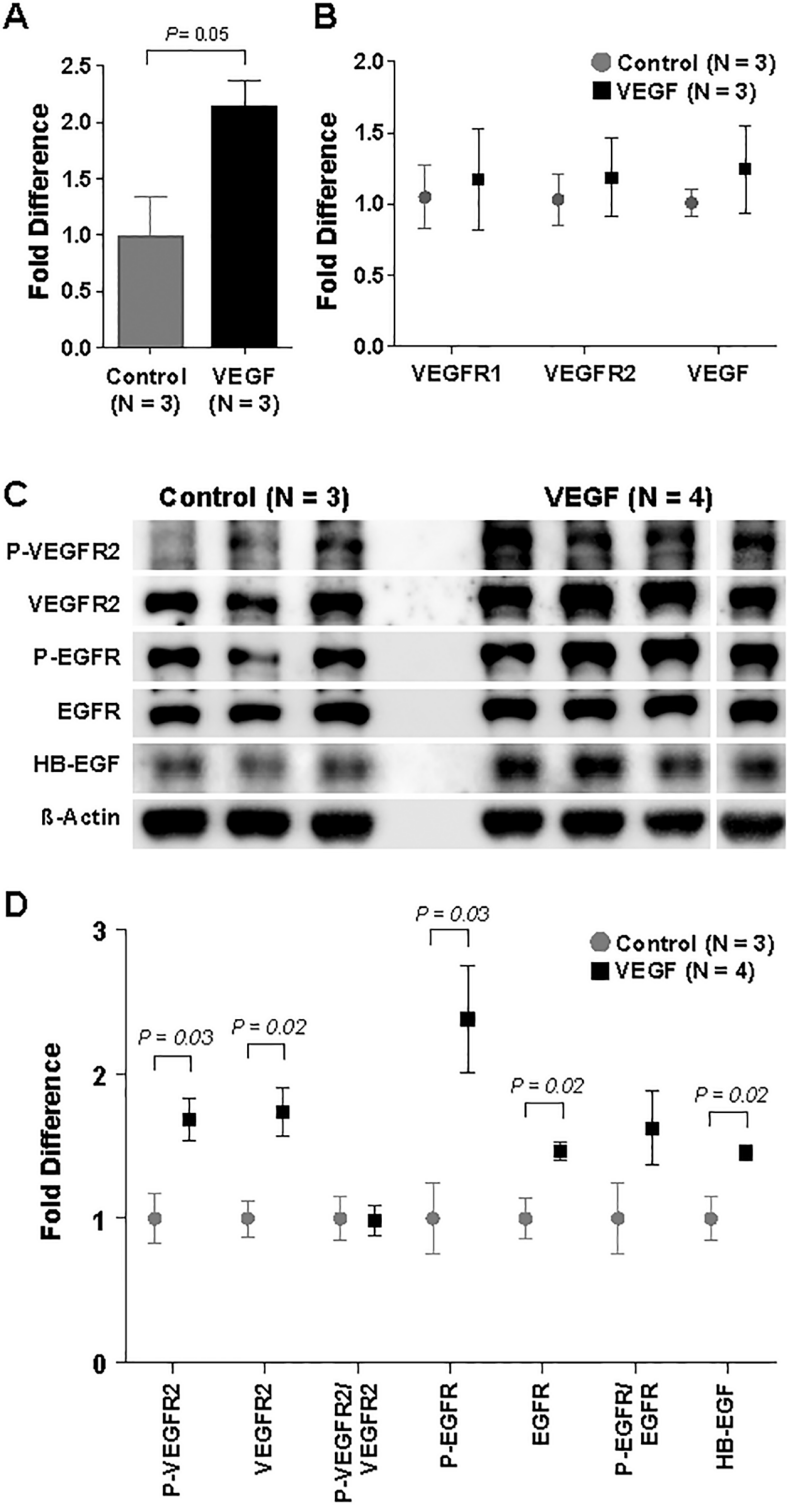
Lung tissue protein expression analyses. ELISA reveals increased VEGF levels in VEGF-treated lungs (A) on POD 4. However, quantitative polymerase chain reactions (qPCR) show no difference in mRNA transcript levels of VEGF, VEGFR1, or VEGFR2 between the two groups (B). Immunoblot demonstrates an increase in the levels of P-VEGFR2, VEGFR2, P-EGFR, EGFR, and heparin-binding EGF-like growth factor (HB-EGF) with VEGF treatment (C-D). Data are expressed as mean ± SE.

In order to determine the effects of daily treatment on VEGF expression, a VEGF ELISA was utilized to quantify the levels of VEGF in lung tissues harvested on POD 4. There was a 2.1-fold increase in VEGF levels in the VEGF-treated lungs as compared to the control group (*P* = 0.048) (Fig 4A). However, on qPCR analyses, there was no difference in the levels of VEGF mRNA transcripts (Fig 4B). Therefore, the difference in VEGF levels between the two groups on ELISA was likely a result of their experimental treatments instead of an endogenous increase in expression of the protein.

Analyses of lung tissues on POD 4 also revealed an increase in activated VEGFR2, P-VEGFR2, in the VEGF group (*P* = 0.03) (Fig 4C and 4D). Interestingly, there was also an increase in total VEGFR2 expression with VEGF treatment (*P* = 0.02). Therefore, there was no difference in P-VEGFR2/VEGFR2 ratio. Since there was no difference in VEGFR2 transcript levels on qPCR (Fig 4B), the increase in VEGFR2 expression on immunoblot could be a result of decreased receptor degradation. Trafficking of VEGF receptors has been shown to be influenced by their interactions with VEGF ligands and the extracellular matrix [31]. Similarly, VEGF treatment resulted in an increase in the levels of both P-EGFR (*P* = 0.03) and EGFR (*P* = 0.02) with no changes in P-EGFR/EGFR ratio. As HB-EGF shedding has been shown to be dependent on VEGFR2 activation [32], its presence was examined in the lungs and found to be significantly increased with VEGF treatment (*P* = 0.02) (Fig 4C and 4D). These data suggested that HB-EGF could be the link between angiogenic stimulation and epithelial cell proliferation in the context of lung growth.

### VEGF stimulated lung epithelial cell proliferation via HB-EGF as a paracrine factor

**Fig 5:**
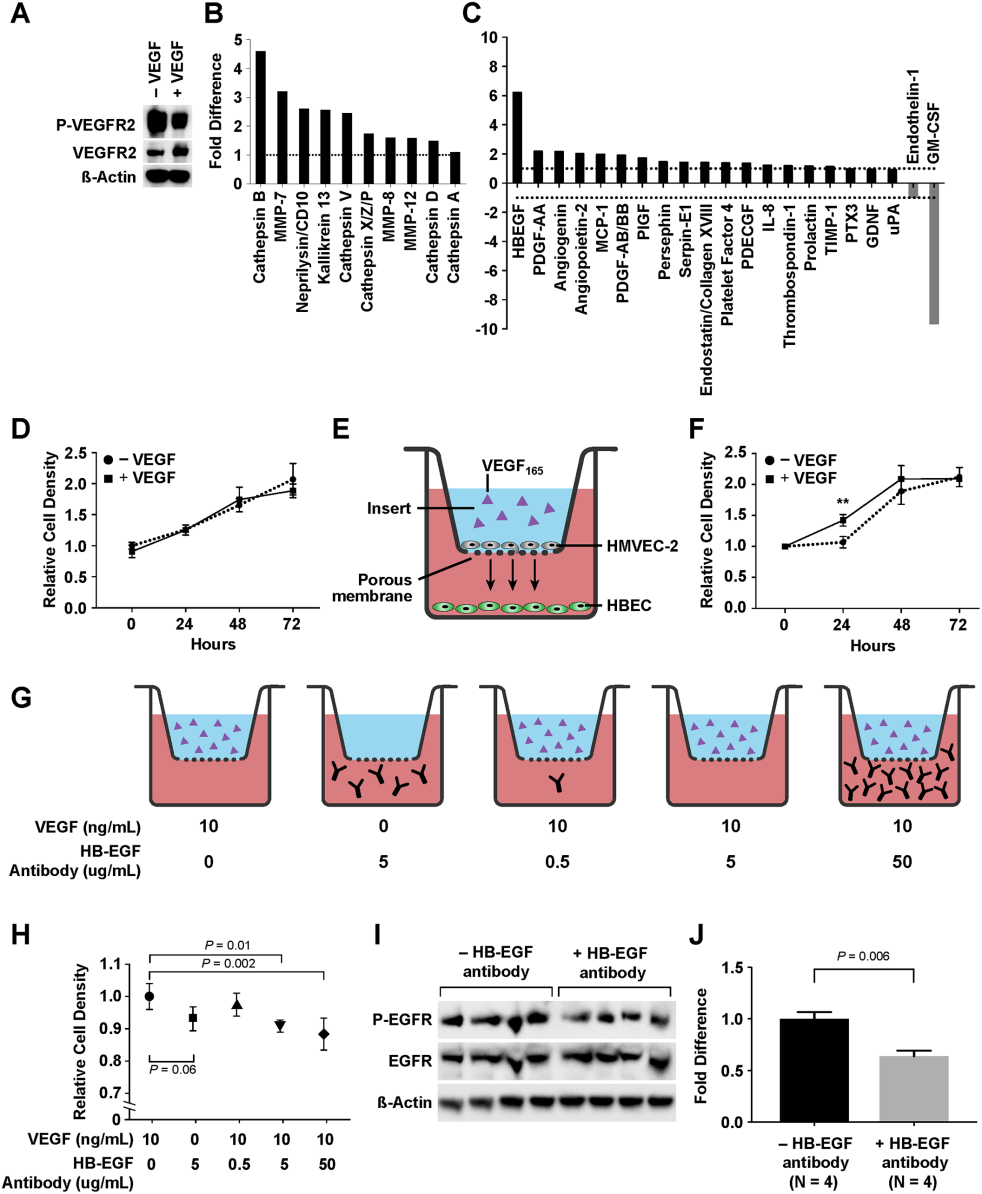
Co-culture assays of human bronchial epithelial cells (HBEC) and human lung microvascular endothelial cells (HMVEC-L). VEGF_165_ at 10 ng/mL activated HMVEC-L (A). HMVEC-L treated with VEGF_165_ show upregulation of proteases such as cathepsin B and V, matrix metalloprotease (MMP)-7, CD10, and kallikrein 13 (B) as well as a more than 6-fold increase in HB-EGF concentration in the conditioned medium (C). HBEC treated directly with VEGF show no increase in proliferation (D). However, in a co-culture model with HMVEC-L (E), proliferation increases when HBEC is co-cultured with VEGF-treated HMVEC-L (F). When an HB-EGF neutralizing antibody is added to co-cultured HBEC (G), proliferation decreases at higher concentrations of the antibody (H). Activation of EGFR on co-cultured HBEC is confirmed to decrease in the presence of an HB-EGF neutralizing antibody (I-J).

In order to further investigate the roles of HB-EGF in mediating the effects of VEGF on lung growth, we performed a series of *in vitro* assays utilizing HMVEC-L and HBEC. First, we confirmed the activity of VEGF_165_ by examining its activation of VEGFR2 on HMVEC-L (Fig 5A). We then characterized the protein contents of HMVEC-L conditioned medium after VEGF stimulation. Compared to untreated cells, VEGF treatment resulted in an upregulation of multiple angiogenic products, most notably a 6-fold increase in HB-EGF concentration (Fig 5C). Analyses of HMVEC-L cell lysate also revealed an increase in the levels of multiple proteases, most notably cathepsin B and V, matrix metalloprotease (MMP)-7, CD10, and kallikrein 13 (Fig 5B). Of these proteases, cathepsin B and MMP-7 have been demonstrated to promote the release of HB-EGF from its transmembrane precursor, a process called ectodomain shedding [33–35]. Data from these assays confirmed that VEGF stimulation of lung endothelial cells resulted in an upregulation of proteases, which in turned increased the shedding of HB-EGF.

We next sought to determine if HB-EGF released from lung endothelial cells could accelerate epithelial cell proliferation. First, we confirmed that VEGF added directly to HBEC resulted in no changes in cellular proliferation (Fig 5D). Next, HMVEC-L and HBEC were co-cultured and allowed to communicate with each other through a porous membrane of 0.4 μm in size (Fig 5E). When VEGF was added to the HMVEC-L chamber, HBEC proliferation was increased at 24 hours of incubation (*P* = 0.002) (Fig 5F). To identify if HB-EGF was indeed responsible for this increase in HBEC proliferation, the assay was repeated with the addition of an HB-EGF neutralizing antibody into the HBEC chamber (Fig 5G). In the presence of VEGF-stimulated HMVEC-L, the HB-EGF antibody at a concentration of 5 and 50 μg/mL significantly decreased HBEC proliferation (*P* = 0.01 and 0.002, respectively) (Fig 5H). Furthermore, examination of HBEC cells treated with and without the antibody confirmed a decrease in activation of EGFR (*P* = 0.006), the main target of HB-EGF [36] (Fig 5I and 5J). Collectively, these assays demonstrate that HB-EGF is an important paracrine factor that links angiogenic stimulation to epithelial proliferation and may mediate the effects of VEGF on enhancing CLG.

## Discussion

Previous work from our group demonstrated the effects of systemic VEGF on accelerating CLG after left PNX [13]. In this study, we sought to replicate these results with topical administration of VEGF. Consideration was given to aerosol inhalation, intratracheal instillation, and nasal instillation. Although aerosol inhalation has been shown to result in a more uniform pulmonary distribution, dosage delivery is variable and limited [37]. Intratracheal instillation requires deep sedation and intratracheal intubation, which is traumatic to the animal if performed on a daily basis. Although nasal instillation presents certain limitations, such as heterogenous intrapulmonary distribution and central nervous system absorption [21], it appeared to be the optimal approach due to the benefits of controlled dosage and light sedation requirement.

Compared to systemic injection, nasal instillation significantly decreased the amount of VEGF absorbed into the bloodstream. However, targeted intrapulmonary administration of VEGF still resulted in improvement in lung volume and alveolar count. Moreover, the increase in endothelial proliferation observed on both POD 2 and 4 provided supporting evidence for the effectiveness of nasal instillation. The clustering appearance of these proliferating endothelial cells may be a result of the heterogenous distribution associated with nasal instillation. However, it should be noted that these clusters were evenly distributed across the entire right lung and there was no predilection for the peribronchiolar region [37]. The improvement in pulmonary distribution could be a result of higher administered volume used in this study (50 μL/20 g).

Furthermore, the increase in lung volume was supported by an improvement in total lung capacity and pulmonary compliance on pulmonary mechanical studies. Since pulmonary compliance is a measure of volume change against applied pressure and total lung capacity is a direct measure of lung volume in live animals, these parameters have been shown to correlate with lung growth and improve during the process of CLG [38]. Despite the lack of statistical significance, there was also an improvement in walking distance, rest time, and basic and fine movements in VEGF-treated mice on POD 10, especially in the post-treadmill measurements. Since exercise intolerance correlates with pulmonary arterial hypertension (PAH) [39] and VEGF can induce nitric oxide (NO) production [40], the effects of VEGF on exercise capacity seen in this study were possibly a product of PAH alleviation. It is also possible that more pronounced and significant differences in exercise tolerance could be seen with more intense exercise regimens or further post-operative time points.

While the mechanism of how VEGF contributes to microvascular endothelial cell proliferation is widely characterized [41–43], how VEGF leads to bronchial epithelial cell proliferation is not well understood. In the lung, VEGF has been shown to increase both endothelial and epithelial cell proliferation of lung explants *in vitro* [44]. Previous studies have suggested that VEGF may act on endothelial cells to produce tissue specific paracrine trophogens, or ‘angiocrine’ factors, that lead to tissue repair [45]. Particularly in the lung, HB-EGF has been identified as a paracrine factor that links VEGFR2 activation to epithelial cell proliferation [46]. Our study again found HB-EGF to be a key factor that was upregulated by VEGF treatment in both the *in vivo* CLG model and in *in vitro* assays. VEGF had no direct effects on HBEC growth, yet co-culture with VEGF-stimulated HMVEC-L resulted in increased HBEC proliferation. HB-EGF was up-regulated in the conditioned medium of VEGF-treated HMVEC-L and neutralization of HB-EGF abolished the mitogenic effects of VEGF on HBEC. These results suggest that HB-EGF is a paracrine factor that relays angiogenic signaling for epithelial cell proliferation.

HB-EGF is a ligand for EGFR and its upregulation has been shown as a key event in the tissue repair and regeneration of many organs ranging from the skin and cornea to liver and gastrointestinal tract [47–50]. It is synthesized as a transmembrane protein called proHB-EGF, which undergoes further proteolytic cleavage to release the mature, soluble HB-EGF, a process called ectodomain shedding [51]. The concurrent increase in multiple proteases, such as cathepsin B and MMP-7 [33–35] in VEGF-treated HMVEC-L further supported the occurrence of ectodomain shedding as a mechanism for HB-EGF upregulation in this study. In their study, Ding et al also found HB-EGF shedding to be a key event after activation of VEGFR2 and inhibition of this process significantly impaired CLG [46]. In our study, we suggested that HB-EGF may play a key mechanistic role in mediating the effects of VEGF on CLG given its upregulation with VEGF treatment and mitogenic properties on lung epithelial cells. However, epithelial cell proliferation is only one of the key steps involved in alveologenesis and additional factors likely contribute to this process.

The major limitation of this study was the lack of time points beyond POD 10 to evaluate the effects of VEGF treatment on lung growth in the late post-operative period. Furthermore, the lack of statistical significance on pulmonary function and exercise tolerance testing renders it impossible to draw clear conclusions regarding the effect of intrapulmonary VEGF administration on functional status. However, VEGF administration post-PNX trended toward improved functional outcome on POD10 and assessment at a later post-operative time point may reveal more pronounced differences.

Dysregulation of the VEGF pathway has been implicated in many neonatal lung diseases from CDH to bronchopulmonary dysplasia (BPD) [52,53]. Both diseases are characterized by a deficiency in alveolar units either through an arrest in pulmonary development (CDH) or destruction of parenchyma from mechanical ventilation and a hyperoxic environment (BPD) [54]. Additionally, the need for mechanical respiratory support in almost all children born with CDH can further deteriorate their pulmonary functions despite the use of lung-protective ventilator strategies. Promising results were seen with experimental VEGF therapy for the treatment of BPD [9]. The evidence of realveolarization seen with different routes of administration of VEGF provided further evidence for its potential application in the treatment of other hypoplastic lung diseases [9,55].

## Supporting information

S1 File Original data including lung volume, pulmonary function tests, physical activity measurements, morphometric analyses, endothelial proliferation assessment, lung protein expression analyses, and *in vitro* assays. (XLSX)
